# Inhibiting Janus Kinase 1 and BCL-2 to treat T cell acute lymphoblastic leukemia with IL7-Rα mutations

**DOI:** 10.18632/oncotarget.25194

**Published:** 2018-04-27

**Authors:** Emilee Senkevitch, Wenqing Li, Julie A. Hixon, Caroline Andrews, Sarah D. Cramer, Gary T. Pauly, Timothy Back, Kelli Czarra, Scott K. Durum

**Affiliations:** ^1^ Cytokines and Immunity Section, Cancer and Inflammation Program, National Cancer Institute, National Institutes of Health, Frederick, MD, USA; ^2^ Comparative Biomedical Scientist Training Program, NIH, Bethesda, MD, USA; ^3^ Michigan State University, East Lansing, MI, USA; ^4^ Department of Veterinary Medicine, University of Maryland, College Park, MD, USA; ^5^ Laboratory Animal Sciences Program, Cancer and Inflammation Program, National Cancer Institute, National Institutes of Health, Frederick, MD, USA; ^6^ Chemical Biology Laboratory, National Cancer Institute, National Institutes of Health, Frederick, MD, USA

**Keywords:** JAK1, BCL-2, T-ALL, ruxolitinib, venetoclax

## Abstract

Acute lymphoblastic leukemia (ALL) is the most common cancer in children. Current chemotherapy is quite toxic in growing children and more directed therapeutics are being sought. The IL-7R pathway is a major driver of ALL and here we evaluate two drugs directed to that pathway using a model of T cell ALL. Mutant gain-of-function IL-7Rα was transduced into an IL-7-dependent murine thymocyte line conferring ligand-independent survival and growth. JAK1 is associated with IL-7Rα and mediates signaling from the mutant receptor. *In vitro*, treating the transformed cell line with the JAK1/2 inhibitor ruxolitinib inhibited ligand-independent signaling and induced cell death. Transfer of the transformed cell line into mice resulted in aggressive leukemia and untreated mice succumbed in about three weeks. Treatment with ruxolitinib incorporated into chow showed a potent therapeutic benefit with reduction in leukemic burden and extension of survival. BCL-2 is an anti-apoptotic downstream mediator of the IL-7R survival mechanism. Venetoclax, an inhibitor of BCL-2, showed activity against the transformed cell line *in vitro* and could be combined with ruxolitinib *in vivo*. These findings support the therapeutic potential of treating T-ALL by targeting the IL-7R pathway.

## INTRODUCTION

Acute lymphoblastic leukemia (ALL) is an aggressive hematological malignancy resulting from the transformation of immature B or T cells and is the most common pediatric cancer. In the 1940’s, the first effective chemotherapy was developed to treat ALL, increasing the cure rate from 10% to 80–90% today. However, the current ALL chemotherapy protocols are associated with both acute and chronic toxic side effects, and despite treatment success, ALL remains the second leading cause of cancer deaths in children [1, 2]. Furthermore, T-ALL, which accounts for 15% of pediatric ALL cases, historically has a poorer prognosis than B-ALL. To improve prognosis and survival, it is essential to understand the underlying molecular mechanisms of T-ALL.

IL7Rα mutations are seen in approximately 9% of pediatric T-ALL patients [3–6]. These mutations often occur in the exon 6 region of the gene and lead to the in-frame insertion of three or four amino acids, one of which is almost always cysteine. The cysteine residue induces homodimerization of IL7Rα chains and results in constitutive activation of JAK1 and STAT5 independent of IL-7, JAK3, and γc [3]. In addition to T-ALL, aberrant JAK/STAT signaling occurs in other hematological malignancies including myeloproliferative neoplasms (MPN) such as polycythemia vera [7], B-ALL [8, 9], acute myeloid leukemia (AML) [10, 11], and lymphoma [12, 13]. Additionally, IL7Rα mutations and JAK1 and JAK3 mutations are also reported in early T cell precursor (ETP) ALL, which historically has a poor prognosis [5]. JAK mutations are also common in high risk B-ALL and “Philadelphia chromosome (Ph)- like” B-ALL, which has a transcriptional profile similar to Ph+ leukemia [8, 14]

Since the discovery that the JAK2 V617F point mutation is responsible for 95% of polycythemia vera cases, there has been increasing interest in developing JAK inhibitors for use in hematological malignancies. The idea of using JAK inhibitors as either monotherapy or in combination with other chemotherapies has become an attractive therapeutic strategy in the era or precision medicine, particularly with mutational profiles that have a poor prognosis [15]. Ruxolitinib, a JAK1 and JAK2 inhibitor, was FDA approved in 2011 for the treatment of MPNs with the JAK2 V617F point mutation [16]. In a mouse model of MPN, oral ruxolitinib is effective in reducing splenomegaly, inflammatory cytokines, eliminating malignant cells, and prolonging survival [17].

For most patients, chronic therapy with ruxolitinib does not lead to molecular or pathological remission, due to a phenomenon termed “persistence”, in which JAK/STAT signaling is reactivated [18]. A potential solution to reducing persistence is to combine multiple targeted therapies to inhibit more than one signaling pathway. One such strategy may be to combine ruxolitinib with a BCL-2 inhibitor. BCL-2 is an anti-apoptotic, pro-survival, mitochondrial protein often overexpressed in lymphoid malignancies and is associated with a poor prognosis [19]. Recently, the FDA approved the use of venetoclax, a specific BCL-2 inhibitor that has strong inhibitory activity in multiple types of leukemia [20–22]. Venetoclax has been shown to inhibit growth of BCL-2 dependent leukemias without targeting platelets. BCL-2 is a downstream target of multiple cellular pathways, including the IL-7 pathway. One consequence of IL7Rα activation is the promotion of cell survival by increasing expression of both BCL-2 and MCL1 [23]. Therefore, targeting BCL-2 could also be beneficial in leukemias with IL7Rα mutations.

The model system used in this study is the D1 thymocyte cell line [24] transformed with a mutant human IL-7Rα derived from a patient sequence. It has been previously shown that expression of mutant human IL7Rα in D1 cells has transforming properties and promotes cell cycle and survival independently of IL-7. Here we demonstrate the efficacy of using ruxolitinib and venetoclax to treat T-ALL with IL-7Rα mutations.

## RESULTS

### D1_hIL7RP1 cells result in rapid morbidity, splenomegaly, and hepatomegaly

A thymocyte cell line engineered to express a mutant IL7Rα has been previously described [3]. In brief, the IL-7-dependent thymocyte cell line D1 was derived from a *p53^−/−^* mouse, and subsequently transduced with retroviral vectors that express a IL7Rα mutation derived from a patient sequence (p.Leu242-Leu243insAsnProCys). This mutation was termed “P1”, and the cell line given the designation D1_hIL7RP1. Mutations in p53 are considered rare in T-ALL, at least at initial diagnosis. Approximately one-third of T-ALL relapse cases have mutations in p53; relapse patients with p53 mutations have a poor clinical outcome and shortened duration of survival. Additionally, 50% of T-ALL cell lines, including Jurkat, contain p53 mutations, suggesting that loss of tumor suppressor function is necessary for the successful creation of leukemia cell lines [25–27]. Previous work has shown the mutant human IL-7Rα in this cell line results in ligand-independent phosphorylation of JAK1 and STAT5, and subcutaneous injection results in solid tumor formation at the injection site, significant tissue infiltration, and morbidity within 20 days.

**Figure 1:**
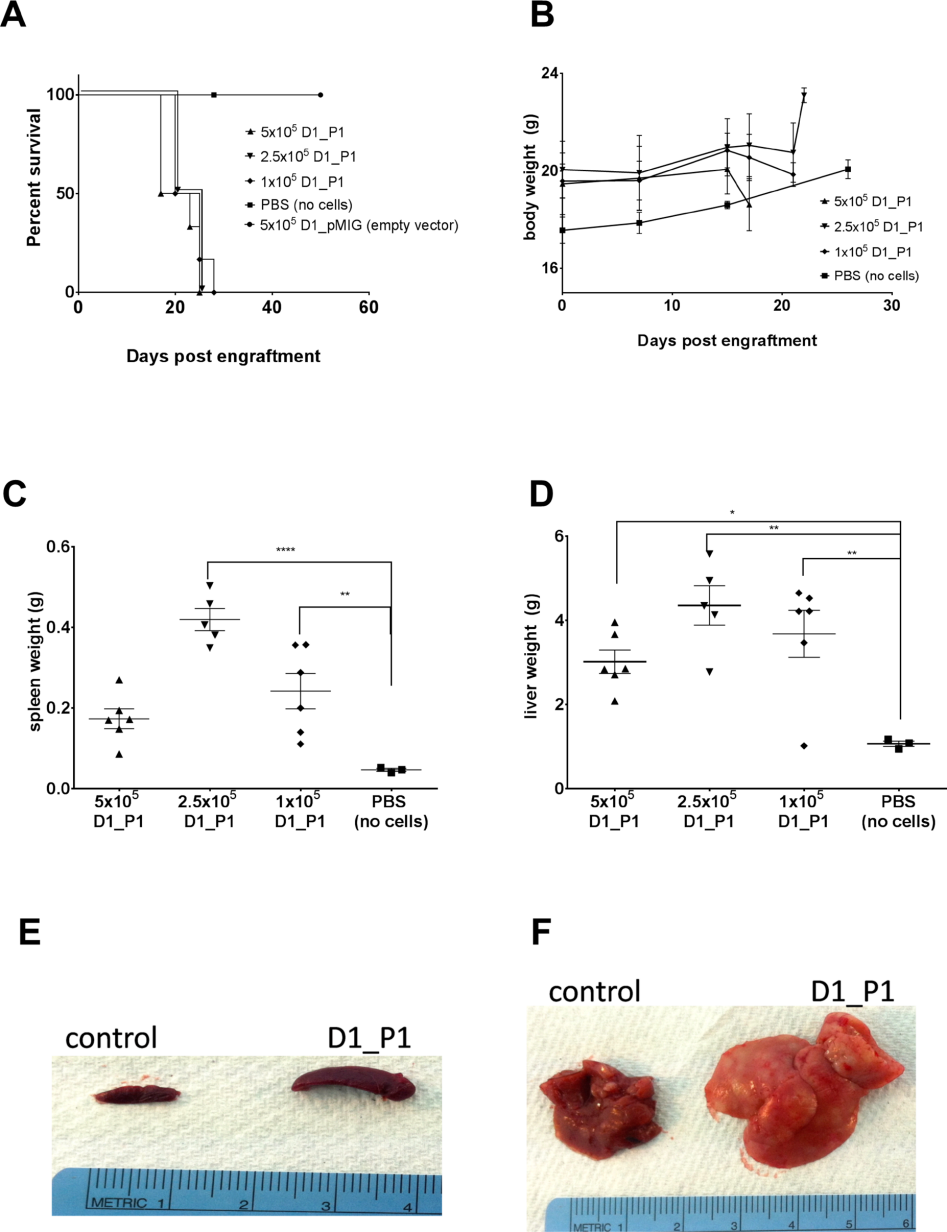
D1_hIL7RP1 cells engrafted into *Rag1*^−/−^ result in rapid morbidity, splenomegaly, and hepatomegaly *Rag1^−/−^* were engrafted with either 5 × 10^5^, 2.5 × 10^5^, 1 × 10^5^, or no D1_hIL7RP1 cells (control, PBS only). (**A**) Kaplan Meier curve depicting survival in days after cell engraftment. (**B**) Weights of *Rag1^−/−^* mice. Mice were to be euthanized if they lost 20% or more of initial body weight. (**C** and **D**) at time of euthanizing, spleen (C) and liver (D) weights were recorded. (**E** and **F**) Representative spleens (E) and livers (F) from mice engrafted with 5 × 10^5^ D1_hIL7RP1 cells (control = no cells, PBS only).

To demonstrate that the D1_hIL7RP1 line could capitulate human ALL, the cells were intravenously injected into sub-lethally irradiated *Rag1^−/−^* mice. Mice were monitored daily for signs of illness, including weight loss, hunched posture, decreased movement, and swollen abdomen as an indication of hepatosplenomegaly. The D1_hIL7RP1 line results in an aggressive leukemia; mice developed clinical signs necessitating euthanasia in 17–24 days depending on number of cells injected (Figure 1A). Loss of p53 alone does not initiate leukemogenesis, as D1 cells transduced with pMIG vector only do not exhibit any signs of leukemia after 50 days (Figure 1A). Leukemic mice did not lose a significant amount of weight during the study (defined as a 20% loss from starting weight) (Figure 1B), but did develop splenomegaly (Figure 1C and 1E) and hepatomegaly (Figure 1D and 1F). We believe the D1_hIL7RP1 begins to replace healthy tissue in the organs, leading to organ failure; notably, the enlarged liver likely obstructs respiration leading to euthanization before extramedullary hematopoiesis develops in the mice receiving 5 × 10^5^ D1_hIL7RP1 cells. For all subsequent experiments, we injected 5 × 10^5^ D1_hIL7RP1 cells, anticipating an experimental time course of approximately 18 days for untreated leukemic mice.

### Ruxolitinib inhibits the proliferation, survival, and STAT5 phosphorylation in D1_hIL7RP1 cells *in vitro*

**Figure 2:**
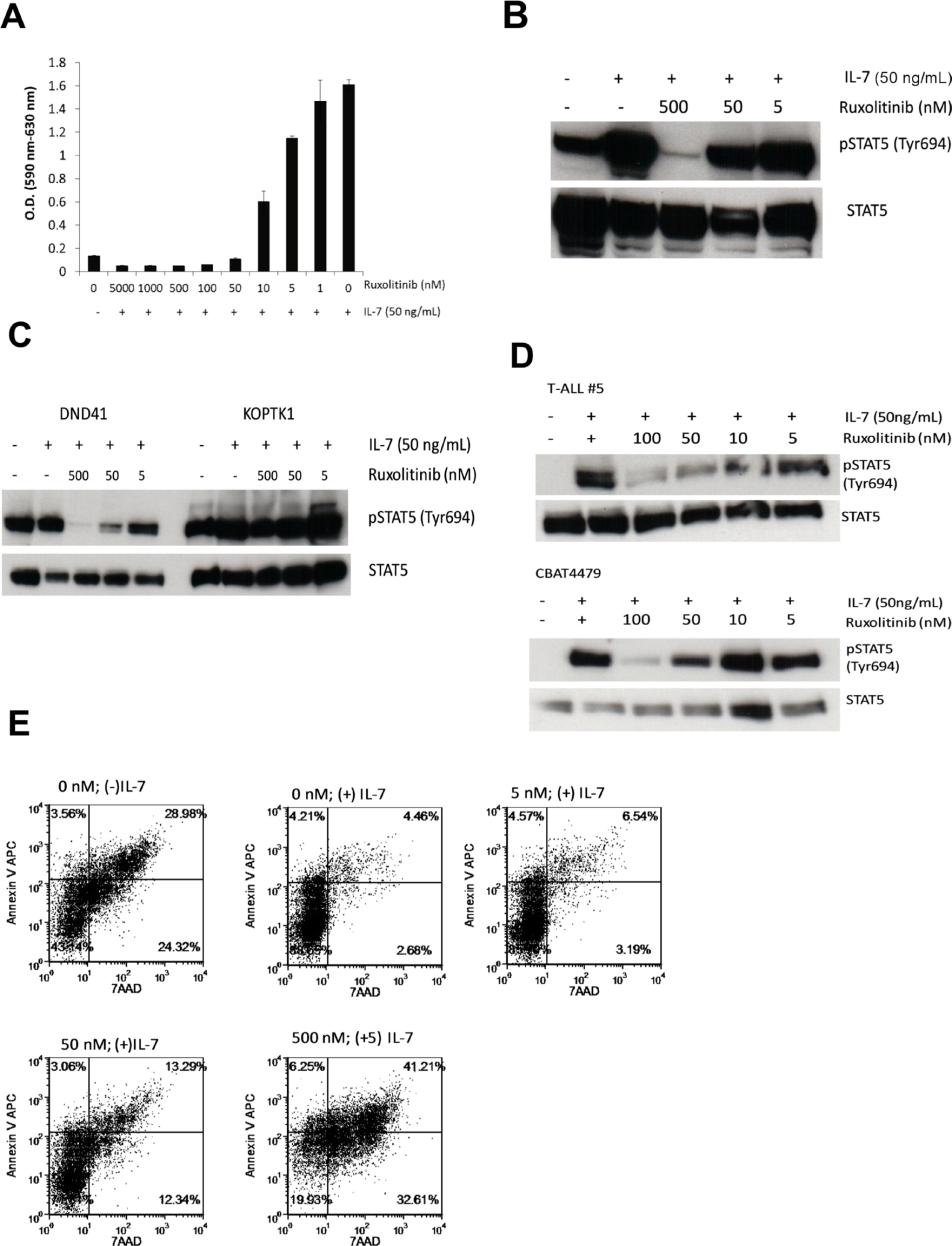
Ruxolitinib inhibits the proliferation and survival of D1_hIL7RP1 cells *in vitro* (**A**) D1_hIL7RP1 cells were treated with ruxolitinib for 48 hours and proliferation was assessed via MTT assay. (**B**, **C**) D1_hIL7RP1 cells (panel B) or human T-ALL *in vitro* cell lines (panel C) were washed, treated with ruxolitinib for 30 minutes and stimulated with IL-7 for 20 minutes. Cells were lysed and phospho-STAT5 levels were assessed via Western blot. (**D**) Human T-ALL cells (passaged in mice) were thawed, rested in media for 1 hour, treated with ruxolitinib for 30 minutes then stimulated with IL-7 for 20 minutes. Cell were lysed and phospho-STAT5 levels were assessed via Western blot. (**E**) D1_hIL7RP1 cells were treated with ruxolitinib for 24 hours and percentage of cells undergoing apoptosis was assessed via Annexin V/7AAD staining. For each FACS plot, the lower left quadrant represents “viable/live” cells while the upper right quadrant represents “dead/late apoptotic” cells.

Next, we evaluated the therapeutic potential of inhibiting the JAK/STAT pathway in cells transformed with mutant IL7Rα. The IL-7 pathway is known to promote T cell survival via induction of antiapoptotic proteins BCL-2 and MCL-1 and proliferation by destabilizing the cell cycle inhibitor p27^kip1^ [28]. Therefore, we hypothesized that blocking this pathway would inhibit the pro-survival and proliferation signals in IL7Rα mutated cells. Ruxolitinib is a potent and selective inhibitor of JAK1 and JAK2 [17] and the mutant IL-7Rα homodimer signals through JAK1 to phosphorylate STAT5. D1_hIL7RP1 cells were treated with ruxolitinib and proliferation was assessed via MTT assay 48 hours later. As shown in Figure 2A, a dose-dependent reduction in proliferation was observed. Ruxolitinib likely affects proliferation by targeting IL7R pathway signaling, as demonstrated by blocking phosphorylation of STAT5 in a dose-dependent manner (Figure 2B). Note that this cell line shows ligand-independent phosphorylation of STAT5 due to the activity of mutant IL-7Rα, although this ligand-independent signaling is weak compared to adding exogenous IL-7, which produces a strong p-STAT5 response (Figure 2A and 2B).

Additionally, similar results can be seen in human T-ALL cell lines passaged *in vitro* and as xenografts in mice. DND41 is an IL-7 independent T-ALL cell line that contains a heterozygous in-frame insertional mutation in exon 6 of IL7Rα [29, 30] similar to D1_IL7RP1. This cell line shows constitutive, IL-7-independent STAT5 phosphorylation that can be blocked with ruxolitinib (Figure 2C). However, another T-ALL cell line KOPTK1 also shows constitutive STAT5 phosphorylation that is resistant to ruxolitinib and IL-7 stimulation, suggesting this cell contains mutations that mediate STAT5 phosphorylation independent of JAK1 (Figure 2C). Other human T-ALL cells such as T-ALL#5 and CBAT4479 that are passaged in immunodeficient mice (cannot be passaged *in vitro*), respond to IL-7 by inducing STAT5 phosphorylation via a pathway that is sensitive to ruxolitinib (Figure 2D).

To demonstrate that inhibition of JAK1 is associated with apoptotic cell death we analyzed the percentage of live and apoptotic cells after 24 hours of ruxolitinib treatment; cells were stained with 7AAD and Annexin V and analyzed by flow cytometry. Treatment of ruxolitinib markedly increased apoptosis compared to cell populations treated with vehicle (Figure 2E). These results demonstrate that ruxolitinib potently impairs the survival and proliferation of D1_hIL7RP1 cells, by inhibiting the IL7R pathway which results in apoptosis.

### Ruxolitinib as a monotherapy has an anti-leukemic effect

**Figure 3:**
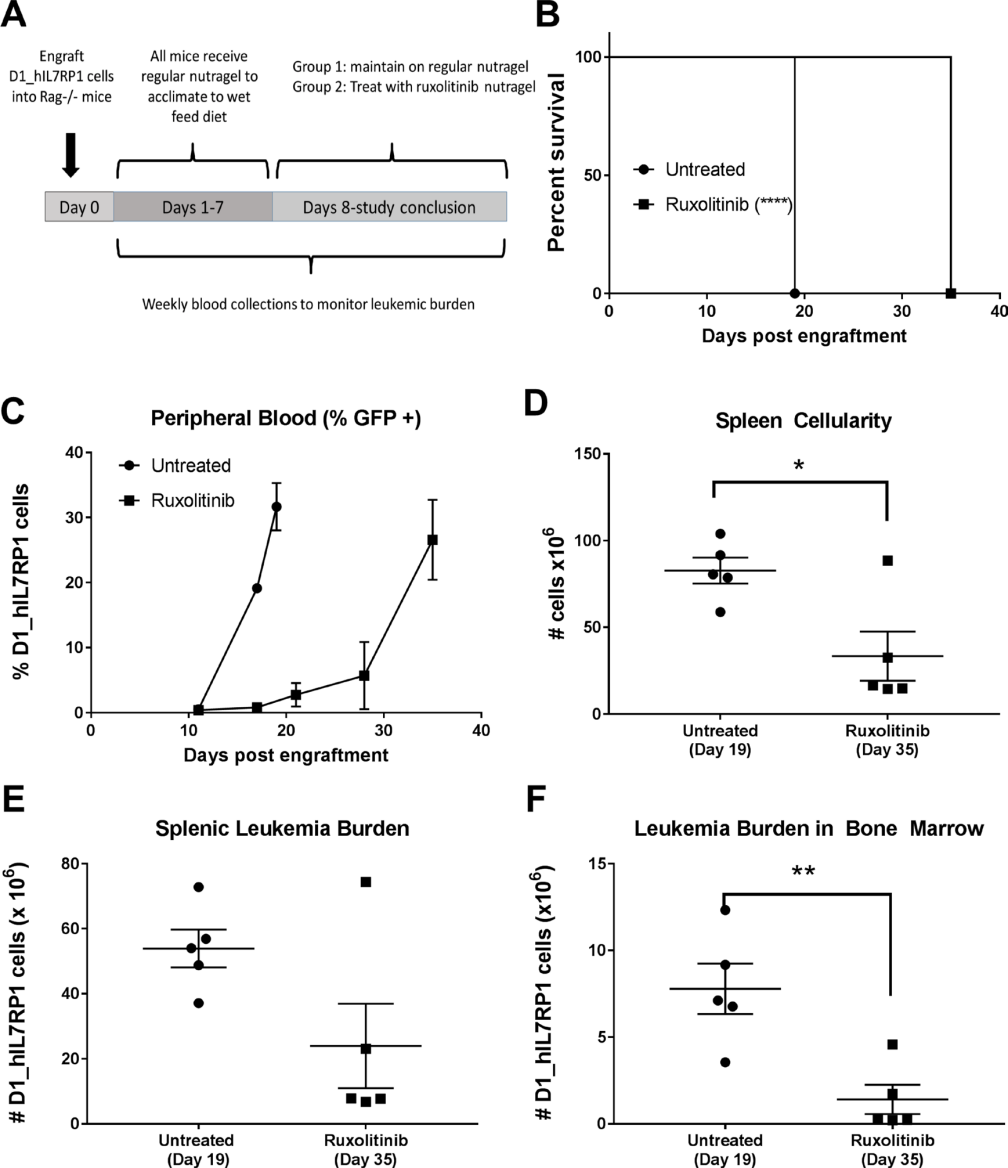
Ruxolitinib delivered via chow is an effective monotherapy (**A**) Treatment schedule. *Rag1^−/−^* mice were engrafted with 5 × 10^5^ D1_hIL7RP1 cells. Treatment was initiated after >1% GFP+ cells were detected by flow cytometry (1 week after cell engraftment). (**B**) Kaplan Meier curve of untreated (*n* = 5) vs. Ruxolitinib treated (*n* = 5) *Rag1^−/−^* mice. (**C**) Peripheral leukemic burden. Blood was collected from a subset of untreated and ruxolitinib treated mice weekly to assess leukemic burden. (**D**) Splenic cellularity of spleens harvested at time of euthanasia. (**E**) Splenic leukemic burden and (**F**) bone marrow leukemic burden assessed by a percentage of GFP+ cells via flow cytometry.

To demonstrate the efficacy of ruxolitinib in leukemia driven by an IL7Rα mutation, we performed therapeutic studies in *Rag1^−/−^* mice engrafted with D1_hIL7RP1. Once mice had sufficient disease burden to detect >1% GFP+ cells in the peripheral blood, a subset of mice were treated with ruxolitinib supplied in nutragel chow (Figure 3A). Leukemia burden was assessed by weekly analysis of peripheral blood and spleen and bone marrow blast percentage at death. Treatment of ruxolitinib increased survival by two weeks (Figure 3B) and slowed progression of leukemic burden in the peripheral blood (Figure 3C). Leukemic burden in spleens at time of euthanasia was reduced in the ruxolitinib group, and spleens were significantly smaller (Figure 3D and 3E) but with equal percentage of leukemic cells as also seen in blood. Leukemic burden in bone marrow was also greatly reduce by ruxolitinib (Figure 3F). Taken together, this indicates that ruxolitinib, even as a monotherapy, can slow the progression of leukemia with an IL7Rα mutation.

### Ruxolitinib can be combined with the BCL-2 inhibitor venetoclax

**Figure 4:**
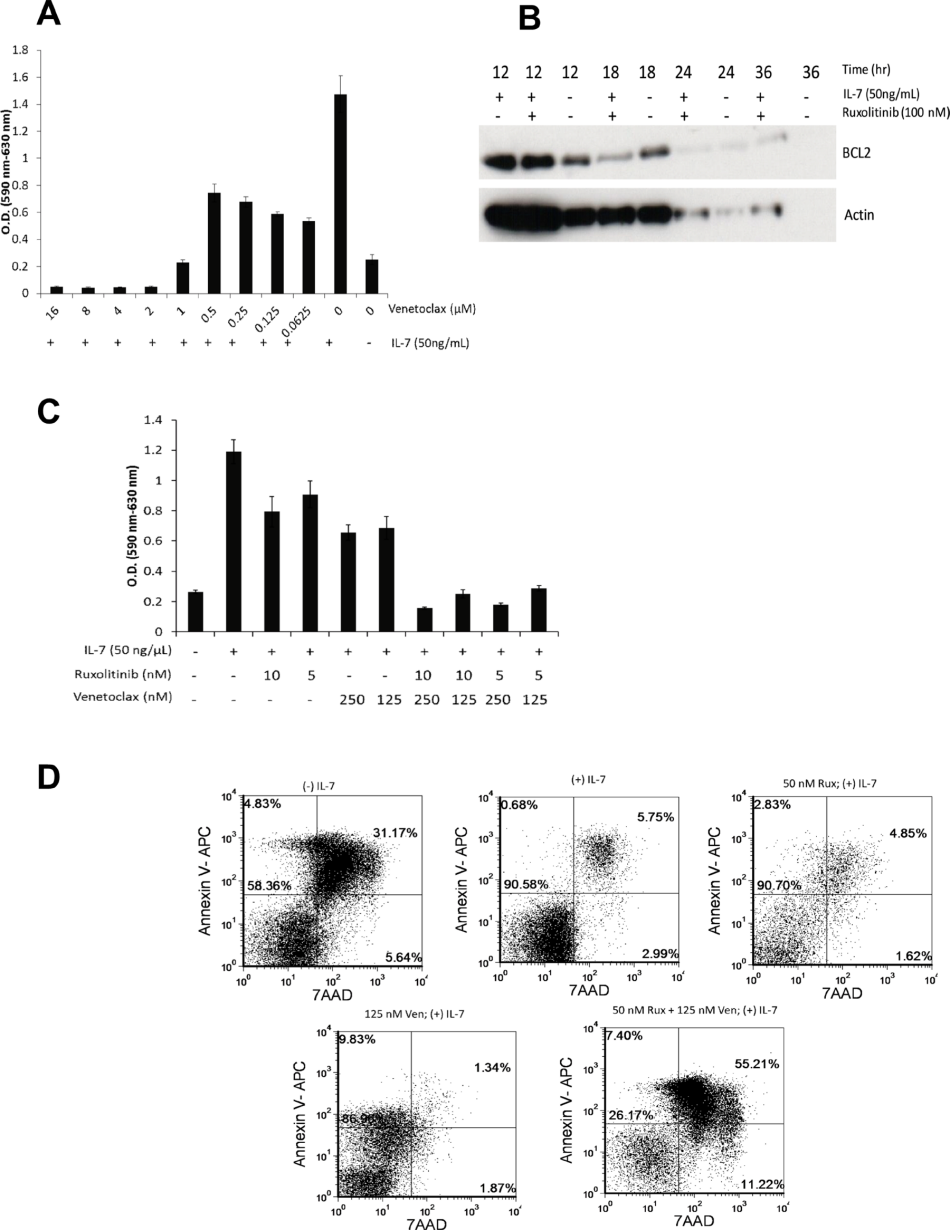
Ruxolitinib and venetoclax are an effective combination *in vitro* (**A**) D1_hIL7RP1 cells were treated with venetoclax alone for 48 hours and proliferation was assessed via MTT assay. (**B**) D1_hIL7RP1 cells were treated with ruxolitinib or starved of IL-7 for 12, 18, 24, and 36 hours. At each time point, cells were lysed and BCL-2 expression was assessed via Western blot. (**C**) D1_hIL7RP1 cells were treated with combinations of ruxolitinib and venetoclax for 48 hours; proliferation was assessed via MTT assay. (**D**) D1_hIL7RP1 cells were treated with ruxolitinib and venetoclax and percentage of cells undergoing apoptosis was assessed via Annexin V/7AAD staining. For each FACS plot, the lower left quadrant represents “viable/live” cells while the upper right quadrant represents “dead/late apoptotic” cells.

**Figure 5:**
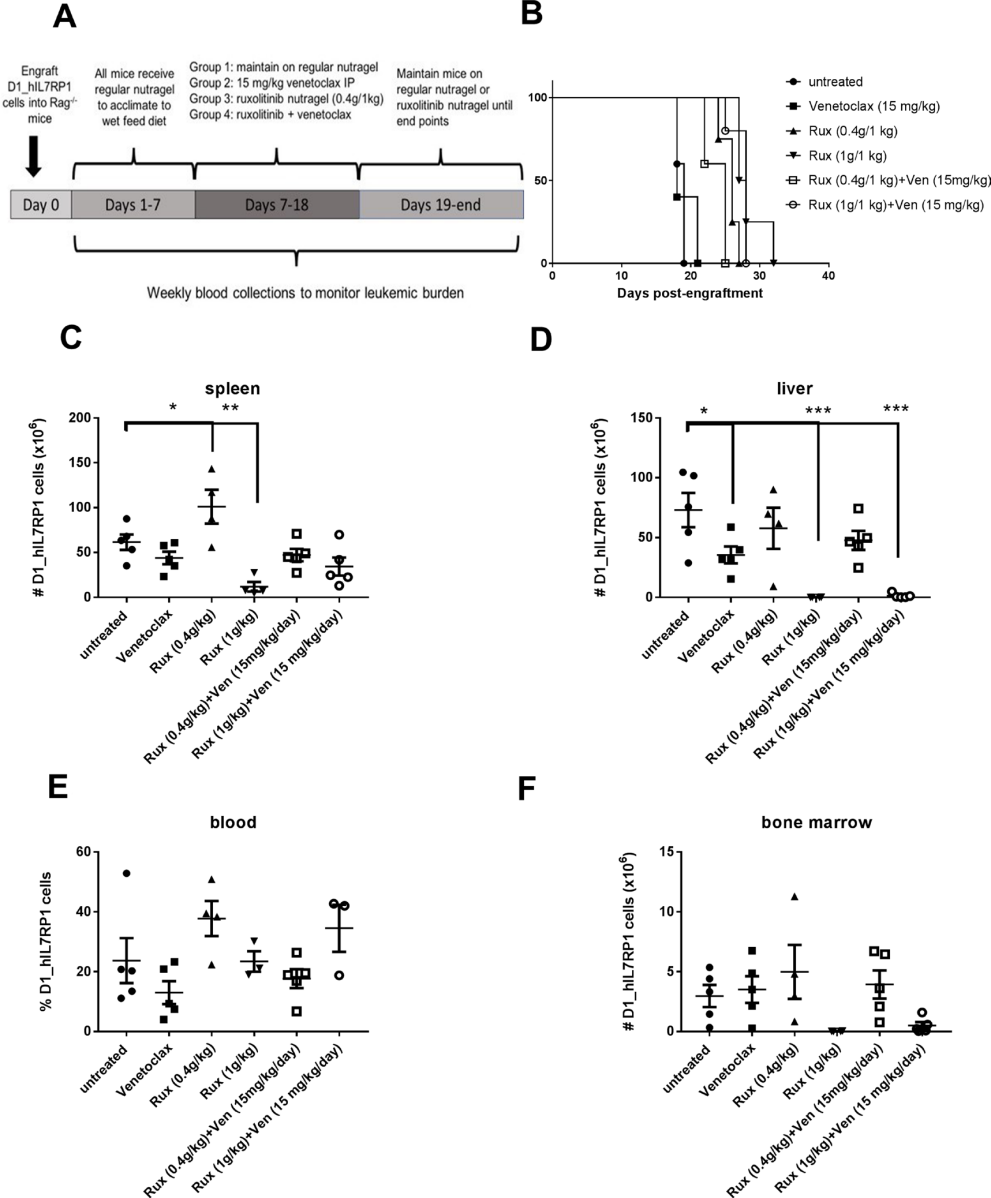
Venetoclax and Ruxolitinib can be used to treat T-ALL *in vivo* (**A**) Treatment schedule. *Rag1^−/−^* mice were engrafted with 5 × 10^5^ D1_hIL7RP1 cells. Treatment was initiated after >1% GFP+ cells were detected by flow cytometry (1 week after cell engraftment). Intraperitoneal treatment of venetoclax was combined with 2 different doses of ruxolitinib nutragel, at a ratio of 0.4 mg/1 kg nutragel and 1 mg/1 kg nutragel. (**B**) Kaplan Meier curve depicting survival in days after cell engraftment. (**C**–**F**) Spleen (C), liver (D), peripheral blood (E), and bone marrow (F) leukemic burden assessed by determining total number of GFP+ cells per organ via flow cytometry.

To determine if venetoclax has potential therapeutic relevance to IL7Rα mutations, D1_hIL7RP1 cells were treated *in vitro* with venetoclax for 48 hours and proliferation was assessed via MTT assay. As shown in Figure 4A, venetoclax was moderately effective as a single agent in inhibiting proliferation. Ruxolitinib is also moderately effective at decreasing BCL-2 expression by 18 hours, suggesting these two drugs could have a combinatorial effect (Figure 4B). Indeed, the combination of ruxolitinib and venetoclax was markedly more effective at inhibiting proliferation (Figure 4C) and inducing apoptosis (Figure 4D) than either drug alone. To determine whether these two drugs could be effective *in vivo,* mice were intravenously injected with D1_hIL7RP1 cells, and then treated with venetoclax alone (by intraperitoneal injection), ruxolitinib alone (given in feed), or both drugs together (Figure 5A). Preliminary studies had shown that combining the two drugs orally resulted in severe gastrointestinal toxicity. Venetoclax alone had no effect in extending survival compared to untreated mice (Figure 5B); however, lower concentrations of ruxolitinib (previously shown in Figure 3) were still effective at extending survival of mice by 8–13 days (Figure 5B). Although there was no benefit of combining the two drugs on survival (Figure 5B) possibly because of toxicity, the drug combinations had variable effects on reducing leukemogenesis. A combination of a low dose of ruxolitinib combined with venetoclax decreased leukemic burden in the spleen (Figure 5C), liver (Figure 5D), bone marrow (Figure 5F), but not in the peripheral blood (Figure 5E). The gastrointestinal toxicity of delivering both drugs orally may be avoidable since we show that delivering venetoclax by intraperitoneal injections in combination with oral ruxolitinib did not result in gastrointestinal side effects (data not shown). This suggests that alternative routes of delivery for venetoclax in humans should be explored.

## DISCUSSION

The IL-7R pathway is a major driver of T-ALL as evidenced by gain-of-function mutations in IL-7Rα, as well as other perturbations in the IL-7R pathway [15]. JAK1 is associated with IL-7Rα, leading us to evaluate efficacy of ruxolitinib, a potent JAK1 inhibitor in a leukemia model driven by mutant IL-7Rα. We observed that ruxolitinib showed anti-leukemic activity *in vitro* and *in vivo* in mice. We also found that venetoclax, a BCL-2 inhibitor, showed anti-leukemic effects *in vitro* that strongly potentiated ruxolitinib activity, and *in vivo* we present data showing the two drugs could be combined, though only ruxolitinib as monotherapy was effective in extending survival. To date, this is the first *in vivo* study demonstrating the effectiveness of combining ruxolitinib and venetoclax to treat T-ALL.

Although mutations in IL-7Rα do not predict poor clinical outcome [3], many subsets of leukemia with mutations in JAK/STAT pathway components have a high risk of relapse and poor prognosis. Therefore, the idea of using JAK inhibitors in a chemotherapy regimen for T-ALL is an attractive strategy in this era of precision medicine. In this study, we describe the efficacy of ruxolitinib in treating T-ALL with IL7Rα mutations. Previous work has demonstrated that a *p53^−/−^* thymocyte cell line expressing mutated IL7Rα mimics T-ALL [3]. Here we show that this cell line, D1_hIL7RP1, injected via tail vein, results in an aggressive leukemia with hepatosplenomegaly (Figure 1), with extramedullary hematopoiesis being a contributing factor. A unique feature of this cell line is the ability to monitor leukemic burden with weekly peripheral blood draws analyzing GFP+ expressing cells. Ruxolitinib was effective in inhibiting the proliferation and activation of D1_hIL7RP1 cells *in vitro* (Figure 2), extending survival, and reducing leukemic burden in *Rag1−/−* mice when administered in chow (Figure 3). The delivery of ruxolitinib via nutragel rodent chow was more effective at reducing leukemic burden and extending survival than gavage delivery (data not shown). Rather than receiving two doses of 150 mg/kg daily, chow delivery ensures mice are receiving treatment every time they eat. Since ruxolitinib has a short half-life [31], our result suggest that lower, more frequent doses would be more effective than two high concentration doses [32]. Additionally, experiments involving patient derived xenografts may require treatment of mice for thirty or more days; in situations where gavaging would not be an optimal or ethical delivery strategy, nutragel delivery of a drug offers an alternative method.

In a mouse model of adult T cell leukemia driven by HTLV1, potent anti-leukemia effects were seen when ruxolitinib was combined with navitoclax, an inhibitor of BCL-xL and BCL-2 [22]. After navitoclax demonstrated dose-dependent thrombocytopenia, navitoclax was re-engineered into venetoclax, which is specific for BCL-2 [19]. In our T-ALL model, we also show that ruxolitinib can be combined with venetoclax, and the combination *in vitro* is more effective than either drug alone (Figure 4). However, *in vivo,* venetoclax was not effective as a monotherapy, but could be safely combined with ruxolitinib when delivered intraperitoneally rather than orally (Figure 4). In experiments not shown, we observed severe gastrointestinal side effects of combining ruxolitinib and venetoclax orally *in vivo*. In a study combining ruxolitinib and venetoclax to treat AML, the combination of drugs had no effect on increasing survival compared to either drug alone; the authors hypothesize this may be due to toxicity [33].

Though FDA approval for ruxolitinib has been considered a breakthrough therapy for MPNs, it is not a curative treatment. Long term follow up studies have shown that for most patients, ruxolitinib does not lead to molecular or pathological remission [34]. This is likely due to a phenomenon called “persistence”, where JAK/STAT signaling is reactivated via heterodimerization between JAK2 and JAK1 or TYK2. Notably, we observed the ability of the D1_hIL7RP1 cells to evade ruxolitinib treatment; though therapy extended the life of leukemic *Rag1^−/−^* mice, ruxolitinib was not curative and eventually peripheral blood leukemic burden matched untreated mice (Figure 3C). There are new JAK inhibitors in development that may reduce persistence [18, 35, 36]. Additionally, another potential solution may be to combine ruxolitinib or other JAK inhibitors with another targeted therapy that inhibits another signaling pathway. JAK inhibitors have already been combined with mTOR and FLT3 inhibitors in ALL and AML [37, 38]. Other models show promise of combining ruxolitinib with a BCL-2 inhibitor [20, 22, 39]; venetoclax is currently in clinical trials to treat additional hematological malignancies beyond CLL, either as a monotherapy or in combination with other chemotherapeutic agents. Our results certainly encourage future clinical studies to explore the potential to combine venetoclax with ruxolitinib to treat leukemia and potentially other hematological malignancies.

The treatment and cure rates of childhood ALL is considered a major medical success of the 20th century; current treatment protocols result in a five-year overall survival rate of up to 90% in pediatric patients. Despite this progress, there is a great need for a more targeted and less toxic approach to treating hematological malignancies in growing children [40]. With an enhanced understanding of the molecular signatures of these cancers, the current challenge for clinicians will be deciding when it is appropriate to supplement the chemotherapy regimen with targeted drugs, or replace conventional therapeutics all together. Data demonstrating the efficacy of targeted therapeutics combined with genomic analysis of cancer patients should aid in better, less toxic chemotherapeutic and biologic protocols. Leukemia is a heterogenous disease, and patients often have unique mutations; however, clinical analysis has demonstrated that JAK/STAT pathway mutations are quite common [40], and the results from this study contribute to the growing body of knowledge that inhibiting JAK activity may be an effective strategy in treating hematological malignancies.

## MATERIALS AND METHODS

### Reagents

Ruxolitinib and venetoclax were purchased from DC Chemicals. For *in vivo* studies, ruxolitinib phosphate was synthesized by the NCI Chemical Biology Laboratory, Chemistry Support Group. The material synthesized in house was isolated as the R enantiomer (98% ee).

### Cells

Creation of D1_hIL7R_P1 cells have been described previously [3]. Briefly, the D1 thymocyte cell line was transfected with the pUC19 plasmid expressing the mutant IL7R sequence derived from “patient 1” and GFP. D1_hIL7RP1 and D1 were maintained in culture media (RPMI-1640 supplemented with 10% FBS, beta-mercaptoethanol, and penicillin streptomycin) supplemented with 50 ng/mL recombinant human IL-7 (PeproTech). DND41 and KOPTK1 were kindly provided by Terry Fry, National Cancer Institute. T-ALL#5 was a gift from Curt Civin, University of Maryland School of Medicine. CBAT4479 was purchased from The Public Repository of Xenografts, Dana-Farber Cancer Institute.

### MTT assay

Cells were seeded at 1 × 10^5^/well and treated with inhibitors for 48 hours. 15 μL of MTT (3-[4,5-dimethylthiazol-2-yl]-2,5-diphenyltetrazolium bromide; 5 mg/ml; Sigma) was added to each well and incubated at 37°C for 4 hours. 100 μl solubilization solution (Promega) was added to each well, and cells were incubated over night at 37°C. Absorbance was measured by spectrophotometry at wavelengths 590 and 630 nm.

### Cell viability assay

Quantitative determination of cell viability was performed using Annexin V-based apoptosis kits and the manufacturer's instructions (R&D systems). Cells were treated with appropriate inhibitors for 24 hours. Cells were then resuspended in binding buffer, stained with APC-conjugated Annexin V and 7-AAD at room temperature for 15 minutes and analyzed by flow cytometry.

### Immunoblotting

Cell lysates were resolved by 10% SDS-PAGE (Invitrogen), protein was transferred onto nitrocellulose membranes and immunoblotted with antibodies against p-STAT5 (Y694) (1:1000), STAT5 (1:1000) (Cell Signaling Technology), BCL-2 (1:5000) (Santa Cruz), or β-actin (1:20000) (Sigma). Immunodetection was performed by incubation with SuperSignal West Dura Substrate (ThermoFisher).

### Mice

*Rag1^−/−^* mice were originally purchased from The Jackson Laboratory. Mice were maintained at the National Cancer Institute (NCI)-Frederick, Maryland. Animal care was provided in accordance with US NIH Animal Use and Care guidelines. Experiments were approved by NCI-Frederick Animal Care and Use committee. All mice used were 6–10 weeks old.

### *In vivo* experiments

D1_hIL7R_P1 cells (1 × 10^5^–1 × 10^6^ per mouse) were injected intravenously via tail vein into sub-lethally irradiated 6–10 week old female *Rag1^−/−^* mice. Mice were treated with SMZ antibiotics (0.08 mg/ml in drinking water) for 3 days prior to irradiation. Overall health and survival was monitored daily and humanely euthanized at the onset of clinical signs (hunched posture, reduced movement and eating, rough hair coat, or dyspnea). Mice were treated with vehicle or drug once cells had engrafted with a disease burden of >1% peripheral GFP+ blasts, on average about seven days following injection of cells.

### Venetoclax delivery

Venetoclax was formulated in 10% DMSO, 30% polyethylene glycol 400 (Millipore), 5% Tween-20 and 5% glucose in water as described [33]. Venetoclax was delivered by intraperitoneal injection once a day at a dose of 15 mg/kg. Mice were treated with either vehicle or drug for a maximum of 10 days.

### Nutragel delivery of ruxolitinib

Ruxolitinib was delivered in chow formulation as described previously. Chow was prepared by combining Nutragel dry mix kit (Bio-Serv) and ruxolitinib in a ratio of 2 g ruxolitinib to 1 kg nutragel [41], 1 g ruxolitinib to 1 kg nutragel, or 0.4 g ruxolitinib to 1 kg nutragel. The chow was refrigerated at –20°C until use; mice were given *ad libitum* access to chow.

### Analysis of leukemic burden in blood and tissues

For weekly measurements of leukemic burden, peripheral blood was collected retro-orbitally. At the termination of each experiment, blood was collected via cardiac puncture. Tissues were dissociated into single cell suspensions. Red blood cells in spleen and peripheral blood were lysed using ACK lysis buffer (Lonza). Cells were counted using an automated hematology analyzer (Sysmex). Cells were fixed in 1% paraformaldehyde. The total number of GFP+ cells per organ were determined by using a FACSCalibur flow cytometer to determine percentage GFP+ cells multiplied by organ cellularity. Data analysis was performed using FCS Express 5 software.

### Statistical analysis

GraphPad Prism version 7 (GraphPad Software, La Jolla, CA) was used to perform all statistical tests. Differences between groups were calculated using an unpaired two-tailed Student's *t*-test or a 1 way ANOVA with Dunnett's multiple comparisons test. Differences were considered significant at *p* < 0.05.
